# Direct Repair without Augmentation of Patellar Tendon Avulsion following TKA

**DOI:** 10.1155/2015/391295

**Published:** 2015-01-06

**Authors:** Ravi Mittal, Nishikant Kumar, Chandrashekhar Yadav, Ashok Kumar

**Affiliations:** ^1^Department of Orthopaedics, All India Institute of Medical Sciences (AIIMS), New Delhi 110027, India; ^2^AIIMS, New Delhi 110029, India

## Abstract

Complications involving the extensor mechanism after TKA are potentially disastrous. We are reporting a case of patellar tendon rupture from tibial tuberosity following total knee arthroplasty. We managed it by direct repair with fiberwire using Krackow suture technique without augmentation. Our long term result has been very encouraging. Our method is a safe and better method of management of patellar tendon avulsion following TKA when it happens without any tissue loss.

## 1. Introduction

Disruption of the extensor mechanism during or after total knee arthroplasty is an infrequent complication and its management is technically challenging. The incidence of extensor mechanism disruption ranges from 0.17% to 2.5% [1, 2]. Extensor mechanism disruption can be differentiated into a proximal extensor mechanism disruption being a quadriceps tendon rupture or a distal extensor mechanism disruption being a patellar tendon rupture [3]. We are reporting a case of patellar tendon rupture from tibial tuberosity following total knee arthroplasty. We are describing a special suturing technique of patellar tendon to tibial tuberosity, follow-up of which has been very encouraging. To our knowledge, no such technique has been reported in literature for patellar tendon rupture following total knee arthroplasty.

## 2. Case Report

A sixty-year-old man underwent right sided total knee replacement for grade II osteoarthritis. The surgery was performed by a midline incision and medial parapatellar approach. A cemented cruciate substituting posterior stabilized implant (Zimmer LPS) was placed. The patella was everted during surgery and resurfacing was done. The patient had an uneventful recovery and could walk comfortably without support four weeks after surgery. After four weeks, he had a sudden sharp pain around knee joint when he was bending over toilet commode. This pain was accompanied with sound in his knee. His gait became stifflegged and needed support to walk. On examination, he had extension lag of 35 degrees and could flex up to 105 degrees. The knee was not warm or erythematous. There was minimal tenderness around patellar tendon and tibial tuberosity. His X-ray did not reveal any abnormality. A diagnosis of patellar tendon avulsion was made and the knee was explored through the same incision.

**Figure 1:**
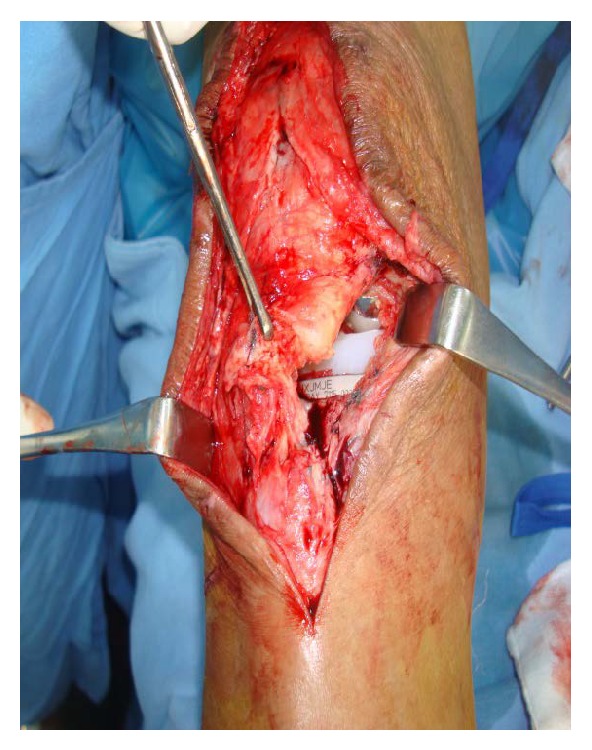
Showing patellar tendon avulsion from distal end with TKA implant in situ.

**Figure 2:**
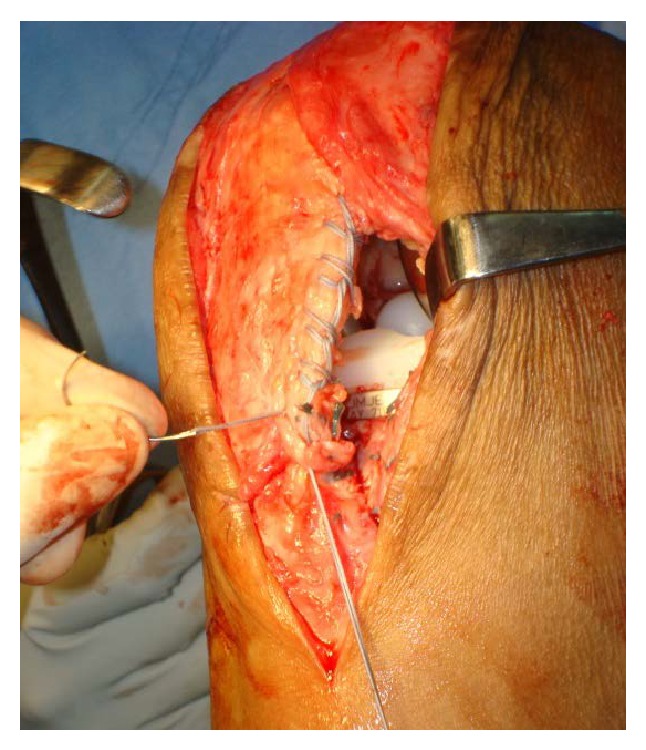
Showing fibre mesh wire placed across patellar tendon (Krackow method).

**Figure 3:**
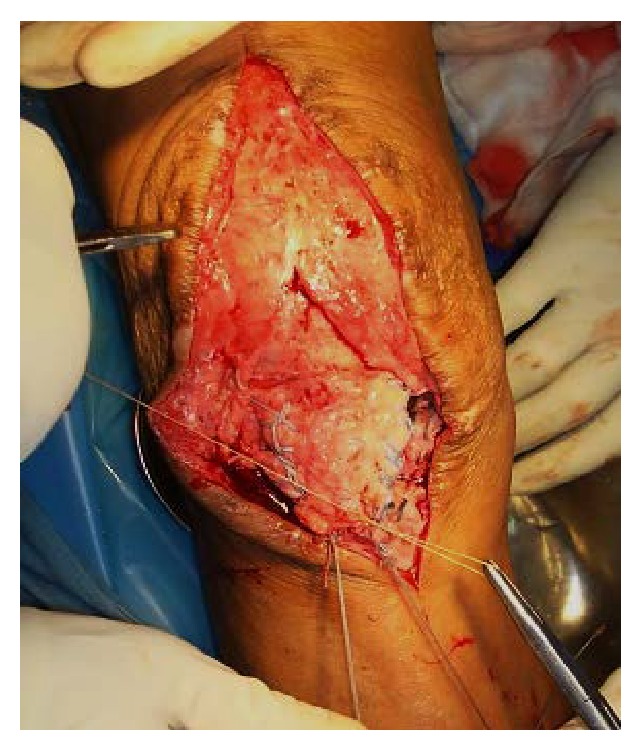
Showing fibre wire suture placed across both ends of patellar tendon.

**Figure 4:**
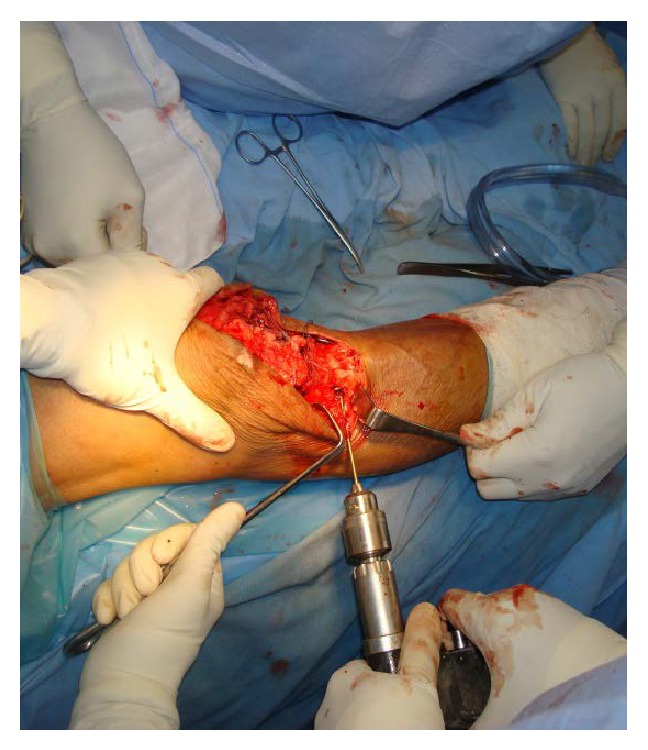
Showing drill being made below tibial tuberosity.

**Figure 5:**
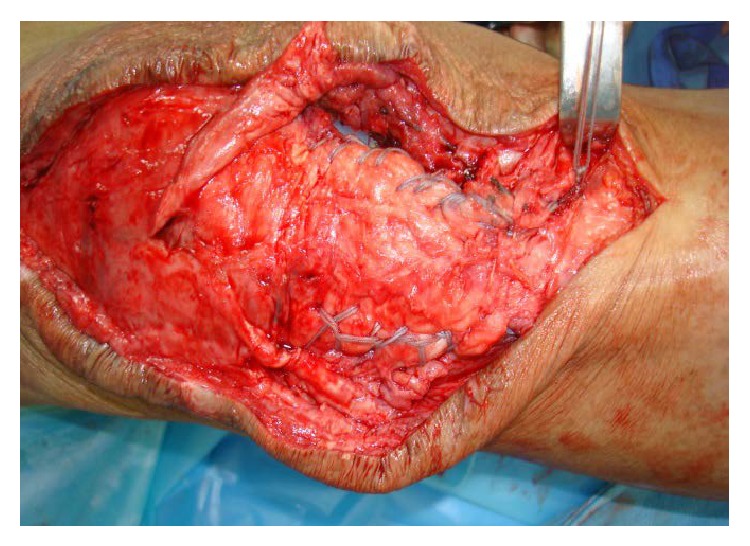
Showing sutures being passed through tunnel and tied.

**Figure 6:**
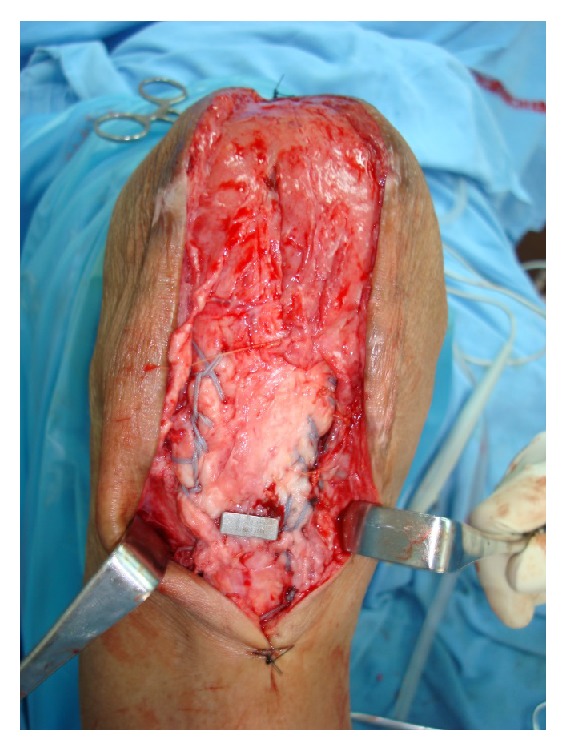
Showing repair being supplemented with staple.

The patellar tendon was found to be avulsed from tibial tuberosity (Figure 1). The tendon was completely detached and a number 2 fiberwire suture was used to apply Krackow suture on each side of the tendon (Figures 2 and 3). A 2.5 mm wide tunnel was drilled 1.5 cm posterior to the tibial tuberosity (Figure 4) and the fiberwire was passed through that tunnel (Figure 5). The two knots were tied on either side of the tuberosity after pulling the tendon up the tuberosity (Figure 5). The reconstruction was further reinforced with a ligament staple (Figure 6) (Arthrex). The wound was closed in layers after hemostasis. The patient was given a cylinder cast for four weeks and allowed full weight bearing. The knee was mobilized after four weeks and dynamic quadriceps exercises were started. He regained 0 to 120 degrees of painless movements without extensor lag.

## 3. Discussion

Falls resulting in extensor mechanism disruption, unless repaired, will lead to disabling loss of knee function. Although these disruptions can occur anywhere along the extensor mechanism, patellar ligament disruptions are likely due to compromise of patellar ligament insertion that follows surgery [1–3].

Treatment for disruption of the extensor mechanism is determined by the level of the disruption (patellar tendon, patella, or quadriceps tendon), degree of functional loss (partial versus full tear), acute versus chronic nature of disruption, availability of viable tissue for either direct primary repair or augmentation, and the health status of the patient [4, 5]. Treatment option includes direct repair alone by suturing, staples or wiring tendons to the tubercle, and primary repair with biologic or synthetic graft or allograft reconstruction of extensor mechanism [1–5].

Decision making in surgical technique depends upon site of rupture, presence or absence of infection, and loss of any tissue from integument. Direct repair can only be done if there is no infection, no loss of overlying integument, and no mid substance rupture and if patellar can be brought down to its original level. Radiological examination may not be useful in presence of surrounding oedema and undisplaced or partial avulsions. However, in complete disruptions, patella alta and bony chips from tibial tuberosity may support diagnosis.

Direct primary repair of a disrupted extensor mechanism with sutures, staples, or wires has shown encouraging results in knees that have not had an arthroplasty. Literature suggests that primary repair rarely restores extensor function after total knee arthroplasty [1, 3, 4, 6]. Dobbs et al. [4] evaluated 18 knees treated for patellar tendon rupture after TKA and reported that direct repair of extensor mechanism has been associated with variable results, with only 25% of the patients having a successful outcome.

The use of synthetic grafts like Dacron and Gore–Tex polypropylene and artificial ligaments like Leeds-Keio ligament allows majority of load during the early postoperative period to be borne by the synthetic graft. Tissue heals and gradually load is transferred to the repaired tissue. Advantages include absence of donor site morbidity. Disadvantages include increased risk of infection and poor results in TKA revisions [7]. Ecker et al. [8] reported a postoperative range of 146.4° using Leeds-Keio ligament to repair either disrupted patellar (6) or quadriceps tendon (5) in eleven patients with one patient having both repaired.

Autogenous tissue graft to augment extensor mechanism reconstruction includes semitendinosus and gracilis tendon, free fascia lata graft, plantaris tendon, or gastrocnemius muscle flap [1, 5, 9]. The semitendinosus and/or gracilis tendon can be placed along the medial border of the patellar tendon, passed through a drill hole in patella using various techniques, and then sutured back on itself under appropriate tension [1, 5, 7]. Noyes et al. [10] evaluated the mechanical properties of human ligamentous graft in young, cadaveric specimens and found that semitendinosus tendon had superior strength compared with graft of gracilis.

If the injury makes primary wound closure difficult, then a medial gastrocnemius flap should be rotated to achieve secure wound closure [9]. Busfield et al. [11] have found that the medial gastrocnemius flap itself can be adequate to restore extensor function.

Augmentation of a disrupted extensor mechanism in the setting of a TKA with an allograft is used to restore the extensor mechanism in a condition where available tissue is mechanically insufficient and structurally inadequate for the demands of knee function [3, 12, 13]. Concerns when using allograft tissue include immune reaction, disease transmission, and graft strength. The risk of immune response has been diminished greatly by deep freezing of the allograft but graft failure is always a concern. Burnett et al. reported promising early results after use of an extensor mechanism allograft to reconstruct a failed extensor mechanism in patients with previous total knee replacement [12, 13]. However, the author concluded that long term results needed further evaluation. The results of Crossett et al. [14] on extensor mechanism repair augmented with Achilles tendon allograft were good and showed improved walking and decreased extensor lag (from 44° preoperatively to 3° postoperatively).

The use of extensor mechanism allograft that includes quadriceps tendon, patella, patellar tendon, and tibial tubercle for reconstruction of a disrupted extensor mechanism was first used by Emerson Jr. et al. [15]. Results using this technique demonstrated improved ambulatory ability, no loss of flexion, and improved extensor lag. Subsequent publications [12, 16] on the results of this technique reported mixed results with poor functional outcome for those cases in which the extensor mechanism allograft was not sutured to the host tissue under maximal tension. Burnett et al. [16] summarized that tensioning the allograft on full extension is a critical determinant of success. The use of allograft for extensor mechanism restoration is best for patients with poor quality host tissue, patients with low functional demand, patients with limited life expectancy, or patients with compromised soft tissue due to multiple previous operations and infections.

It is always better to prevent these hazardous complications from occurring. Intraoperatively, dissections around patellar tendon should be done carefully to avoid disrupting the inserting fibres. In cases of stiff or inflammatory knees, it is better to place a smooth pin or staples around insertion site prophylactically.

The possible cause of patellar tendon rupture in our case could be attributed to the inherent weakness of the tendon due to old age, associated microtrauma, and increased stretch during surgery. Finally, the sudden jerky movement to get up from a low lying commode chair leading to violent contraction of quadriceps might have been the main reason for the subsequent rupture.

Our method of repair is unique and, till our last follow-up (6 years), the patient has comparable ROM to preoperatively. There was no extensor lag. This was probably due to the fact that there was no tissue loss and no infection and optimum tension of the tendon could be restored easily.

Unfortunately, large clinical series that establish the efficacy of any technique do not exist. Our method of repair is unique in the sense that it is simple and easily reproducible and current long term follow-up has shown encouraging result. Our method has a limitation that it cannot be reproduced in situation of chronic patellar tendon rupture tissue loss and infection.

## 4. Conclusion

Complications involving the extensor mechanism after TKA are disabling. Meticulous surgical technique should be used in any patients undergoing TKA in an attempt to prevent these complications from ever happening. Repair using fiberwire in Krackow fashion and passing it through a tunnel beneath tibial tuberosity with stapler is a safe and good method of patellar tendon avulsion from tuberosity when it happens without any tissue loss.
